# Complete Genome Sequence of Salmonella enterica Serovar Typhimurium Siphophage Seabear

**DOI:** 10.1128/MRA.01160-19

**Published:** 2019-10-24

**Authors:** Ketki Patil, Chi Zeng, Chandler O’Leary, Lauren Lessor, Rohit Kongari, Jason Gill, Mei Liu

**Affiliations:** a Center for Phage Technology, Texas A&M University, College Station, Texas, USA; b School of Biology and Pharmaceutical Engineering, Wuhan Polytechnic University, Wuhan, China; Queens College

## Abstract

Salmonella enterica serovar Typhimurium is a foodborne pathogen that causes gastroenteritis. Due to increases in antibiotic resistance, bacteriophage therapy may be an alternative method for preventing *Salmonella* foodborne infections. We report here the complete genome sequence of a T5-like phage, Seabear, which was isolated against S. Typhimurium.

## ANNOUNCEMENT

Salmonella enterica serovar Typhimurium is a Gram-negative bacterium known to cause acute gastroenteritis leading to diarrhea, vomiting, fever, and abdominal cramps (1, 2). Increasing drug resistance found among strains of *S.* Typhimurium has made phage therapy a control measure for *S.* Typhimurium (3, 4). In this paper, we report the genomic characterization of a T5-like siphophage that infects *S.* Typhimurium.

Phage Seabear was isolated from a wastewater treatment plant in College Station, TX, in 2015 using a poultry isolate of *S.* Typhimurium as the host. Host bacteria were cultured on tryptic soy broth or agar (Difco) at 37°C with aeration. Phage were cultured and propagated using the soft-agar overlay method (5). Seabear was identified as a siphophage using negative-stain transmission electron microscopy performed at the Texas A&M University Microscopy and Imaging Center, as described previously (6). Phage genomic DNA was prepared using a modified Promega Wizard DNA cleanup kit protocol, as described previously (6). Pooled indexed DNA libraries were prepared using the Illumina TruSeq Nano low-throughput (LT) kit. The sequence was obtained from the Illumina MiSeq platform using the MiSeq V2 500-cycle reagent kit, following the manufacturer’s instructions, producing 477,731 paired-end reads for the index containing the phage Seabear genome. FastQC 0.11.5 (https://www.bioinformatics.babraham.ac.uk/projects/fastqc/) was used to quality control the reads. The reads were trimmed with FASTX-Toolkit 0.0.14 (http://hannonlab.cshl.edu/fastx_toolkit/download.html) before being assembled using SPAdes 3.5.0 (7). Contig completion was confirmed by PCR using primers (5′-AAACCACTACCCGCACCAC-3′ and 5′-CAGTGAGAAAACGCCGTGTA-3′) facing off the ends of the assembled contig and Sanger sequencing of the resulting product, with the contig sequence manually corrected to match the resulting Sanger sequencing read. GLIMMER 3.0 (8) and MetaGeneAnnotator 1.0 (9) were used to predict protein-coding genes with manual verification, and tRNA genes were predicted with ARAGORN 2.36 (10). Rho-independent termination sites were identified via TransTerm (http://transterm.cbcb.umd.edu/). Sequence similarity searches were done by BLASTp 2.2.28 (11) against the NCBI nr, UniProt Swiss-Prot (12), and TrEMBL databases. InterProScan 5.15-54.0 (13), LipoP (14), and TMHMM v2.0 (15) were used to predict protein function. All analyses were conducted at default settings via the CPT Galaxy (16) and Web Apollo (17) interfaces (https://cpt.tamu.edu/galaxy-pub).

Seabear was assembled at 57.7-fold coverage into a 112,472-bp genome with an 8,522-bp direct terminal repeat, as determined by PhageTerm (18). Seabear has 40.4% GC content, which is lower than that of its host (52.2%) (19). A total of 178 protein-coding sequences and 29 tRNAs were annotated. Genes with assigned functions included those involved in phage morphogenesis, such as major capsid protein, head decoration protein, portal protein, prohead protease, baseplate hub protein, tail tube protein, tail terminator protein, and tape measure protein, and genes involved in DNA replication, such as DNA primase, NAD-dependent DNA ligase subunits A and B, DNA polymerase, and DNA helicase. Lysis genes coding for class III holin, endolysin with a peptidase domain, and an overlapping spanin pair were also found. Seabear shares 94.8% and 59.2% overall DNA sequence identity with *Salmonella* phage Stitch (GenBank accession number KM236244) (20) and phage T5 (GenBank accession number AY543070), respectively, as determined by BLASTn.

### Data availability.

The genome sequence of phage Seabear was submitted to GenBank as accession number MK728824. The associated BioProject, SRA, and BioSample accession numbers are PRJNA222858, SRR8771452, and SAMN11234143, respectively.
